# Pancreas—Its Functions, Disorders, and Physiological Impact on the Mammals’ Organism

**DOI:** 10.3389/fphys.2022.807632

**Published:** 2022-03-30

**Authors:** Monika Karpińska, Marian Czauderna

**Affiliations:** The Kielanowski Institute of Animal Physiology and Nutrition, Polish Academy of Sciences, Warsaw, Poland

**Keywords:** pancreas, pancreatic disorders, pancreatic exocrine insufficiency (EPI), obesity, diabetes

## Abstract

This review aimed to analyze the scientific literature on pancreatic diseases (especially exocrine pancreatic insufficiency). This review also describes the correlation between the physiological fitness of the pancreas and obesity. The influence of the pancreatic exocrine function on the development of the organism of adults and adolescents was also described. The results of piglet studies available in the literature were cited as an established model used to optimize treatments for pancreatic diseases in humans. The pancreas has an exocrine and hormonal function. Consequently, it is one of the key internal organs in animals and humans. Pancreatic diseases are usually severe and particularly troublesome. A properly composed diet and taken dietary supplements significantly improve the patient’s well-being, as well as the course of the disease. Therefore, a diet and a healthy lifestyle positively affect maintaining the optimal physiological efficiency of the pancreas.

## Introduction

The pancreas is a glandular organ that affects the functioning of the entire body. The emerging pancreatic insufficiency is the inability of the pancreas to biosynthesize and/or secrete digestive enzymes in an amount sufficient to digest and absorb food components in the intestines. Insufficiency usually occurs as a result of damage to the pancreas, which can be caused by a variety of clinical conditions, e.g., recurrent acute pancreatitis, chronic pancreatitis, diabetes, autoimmune diseases, after pancreatectomy surgery. It happens that such failure is the result of pancreatic or gastrointestinal cancer. In children, it is most often associated with cystic fibrosis (about 90% of patients) or a rare genetic disease like Shwachman-Diamond syndrome. Pancreatic insufficiency is usually manifested by malabsorption, malnutrition, avitaminosis, and weight loss (or failure to gain weight in children). Treatment is about treating the root cause, preventing further damage to the pancreas, and relieving symptoms.

In this paper, the authors present the impact of a malfunctioning pancreas on the organisms of higher mammals. Based on the analysis of the literature, the influence of the pancreatic exocrine function on the development of the adult and adolescent organism was described. Individual diseases that directly affect the health of the pancreas are listed and briefly described. The correlation between pancreatic diseases and factors such as diet, lifestyle, and obesity was also discussed.

## Structure of the Pancreas

**FIGURE 1:**
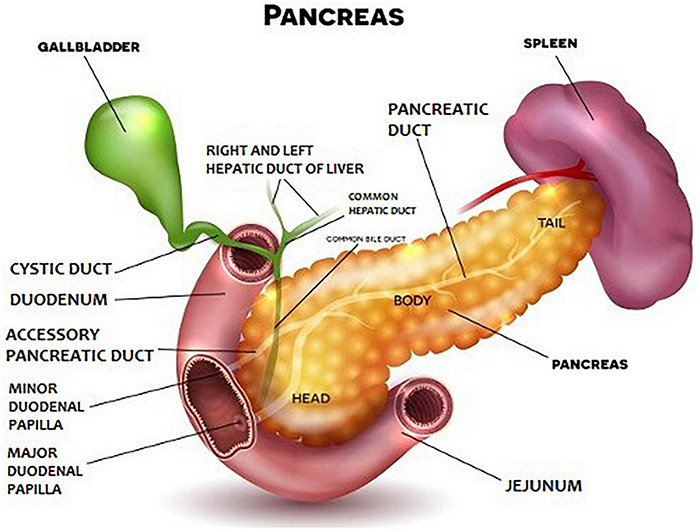
General diagram of the human pancreas (shutterstock).

Anatomically, the pancreas is divided into head, body, and tail (Figure 1). The pancreatic parenchyma has a lobular structure and contains numerous secretory vesicles, which make up 80–85% of the organ’s mass. The discharge ducts are very important for the functioning of the pancreas. Each bubble has an outgoing wire that connects to the others and connects to the main duct. The main duct is the pancreatic duct, which begins in the tail of the pancreas, runs the entire length of the organ, and eventually enters the duodenum through the greater papilla (Vatera). Apart from it, there is also the accessory pancreatic duct, which in about 70% of people connects to the pancreatic duct, and finally, the substance secreted by the pancreas, transported through both ducts, goes to the so-called greater duodenal papilla. In the histological structure of the pancreas, two basic elements are distinguished: pancreatic islets (or Langerhans islands – their number may even reach 2 million and they produce pancreatic hormones) and secretory cells, which constitute the rest of the organ and are responsible for the secretion of pancreatic juice and pancreatic enzymes.

## Physiological Functions of the Pancreas

The pancreas has two essential and very important functions in the body: endocrine (production of hormones that regulate blood sugar levels and glandular secretion) and exocrine (the function of the digestive gland) (Yamada et al., 2005). Endocrine activity is performed by the Langerhans islets and involves the production of hormones such as insulin, proinsulin, amylin, C-peptide, somatostatin, pancreatic polypeptide (PP), and glucagon. Insulin helps to lower blood sugar, and glucagon causes blood sugar to rise. On the other hand, the exocrine activity consists of the production of enzymes that are part of the iso-osmotic, alkaline pancreatic juice and support the digestion of food in the intestines. The intravesical cells produce the enzymatic components of the juice, which is led into the duodenum through the pancreatic ducts. In addition, mucus is secreted in the pancreatic ducts through goblet cells. The composition of pancreatic juice includes enzymes that digest proteins, fats, carbohydrates, and nucleic acids, as well as electrolytes and a small amount of mucus (Dąbrowski et al., 2007).

Enzymes such as trypsin, chymotrypsin, carboxypeptidase, and elastase belong to the group of proteolytic enzymes (they digest proteins). Trypsin and chymotrypsin are secreted in the form of proenzymes: trypsinogen, chymotrypsinogen. The pancreatic lipolytic enzymes are lipase, phospholipase, and esterase, which digest fats. The glycolytic (carbohydrate digesting) enzymes are lactase and amylase, which breaks down starch into maltose, maltotriose, and dextrins. Nucleolytic enzymes include ribonuclease and deoxyribonuclease, which break down nucleic acids into mono- and oligonucleotides. Food consumption and the neurohormonal mechanism regulate the secretion of digestive enzymes. The pancreas secretes pancreatic juice in a volume of about 1–4 liters per day, and this amount depends on the food consumed.

## Influence of the Exocrine Function of the Pancreas on the Development of the Adult and Adolescent Organism

The central nervous system and hormones regulate the exocrine function of the pancreas. Hormones such as secretin and cholecystokinin (CCK) are believed to be the main intestinal hormones that regulate the secretion of pancreatic digestive enzymes (Morisset, 2020). Secretin is released from enteroendocrine cells in the small intestine, and CCK is released from the duodenum and jejunum in the presence of lipids and proteins from ingested food. Kapica et al. (2018) described the effect of obestatin (a hormone produced in specialized cells in the gastric and small intestine of many animals, including humans) on the exocrine pancreatic secretion in rats. They found that this hormone can stimulate the secretion of pancreatic juice through two opposing mechanisms.

For a long time, scientists have been trying to better understand the impact of the physiological functions of the pancreas on the human body. The recognized model closest to humans is the pig model. CCK has also been shown to be the main regulator of the exocrine function of the pancreas in pigs, despite the lack of CCK receptors (Schweiger et al., 2000; Morisset et al., 2003). It has also been observed that pancreatic development in pigs appears to be more dependent on diet change at weaning than on age (Pierzynowski et al., 1993). The consumption of milk by a piglet causes a postprandial rise in glucose, but not in insulin (Pierzynowski et al., 1995). Therefore, it can be concluded that milk can regulate the exocrine function of the pancreas by producing the amount and type of enzymes needed for digestion. In the available literature, some reports indicate a positive correlation between the exocrine function of the pancreas and weight gain in both suckling piglets (van den Borne et al., 2007) and in young piglets (Botermans and Pierzynowski, 1999; Pierzynowski et al., 2005). Pierzynowski et al. conducted an experiment on piglets, in which in 7 out of 10 pigs tested, with increasing body weight, they observed an increase in exocrine pancreatic secretion and a higher exocrine pancreatic secretion after a meal than preprandial secretion (Flegal et al., 2010). Disturbances in digestion and feed absorption in pigs with EPI result in slower growth of the animal. However, as pigs age, the impact of the pancreatic exocrine function on organism growth decreases. Gregory et al. found that in pigs of ∼ 30 kg body weight, EPI causes complete stunting (Gregory et al., 1999), and Corring and Bourdon (1977) only observed a 25% growth retardation in pigs ligated at 40 kg. Pierzynowski et al. (1990) conducted studies on the development of pancreatic exocrine function in piglets. The experiment consisted in monitoring gastric secretion and the excretion of total protein and trypsin before and after feeding during the 1st 13 weeks of animal life (Pierzynowski et al., 1990). The results of the studies showed that the basal pancreatic function and secretory response to feeding remained low until 4–5 weeks of age. Only after weaning the piglets, both the secretion of pancreatic juice and the secretion and activity of trypsin increased significantly. It was also observed that the enzymatic composition of pancreatic juice changed qualitatively during this period. Moreover, intravenous administration of cholecystokinin (CCK) and secretin did not stimulate exocrine function during the 1st 2 weeks of age, while a significant effect was observed from 3 to 4 weeks of age. Thus, during individual development, they found an increase in the exocrine pancreatic function and a qualitative change in the pattern of hydrolytic enzymes. They also observed an increase in the pancreatic response to hormonal stimulation during the feeding period.

Fedkiv et al. (2009) investigated the growth performance of pigs with exocrine pancreatic insufficiency (EPI) at different ages. They subjected the experiment to twelve 7-week-old weaners and twelve 16-week-old pigs during the fattening period. Six pigs from each group underwent surgery for ligation of the pancreatic duct. They monitored the growth of the animals and recorded the consumption of porcine pancreatin enriched feed (Creon^®^ 10000). They observed that EPI caused growth inhibition in weaner pigs but did not affect the growth of older pigs compared to the corresponding unoperated groups of pigs. Older pigs subjected to the experiment showed the ability to adapt to EPI without apparent negative effects on their growth. In addition, pancreatin feed supplementation has been shown to increase feed consumption (i.e., improve appetite) in both pigs with EPI and non-operated pigs.

In another experiment, Rothenbacher et al. measured the amount of pancreatic elastase-1 in their stools in a large group of older people (i.e., 914 people aged 50–75. In this group, 11.5% of respondents showed signs of EPI, and signs of severe exocrine pancreatic insufficiency (SEPI) were shown by 5.1% of surveyed people (Rothenbacher et al., 2005). The incidence of both diseases was greater in men than in women. Researchers found a marked increase in EPI and SEPI with the age of patients. Then, the influence of various factors on the occurrence of EPI and SEPI was analyzed. It was found that those consuming less alcohol had the lowest incidence of both diseases (EPI 7.4%, SEPI 3.1%) than those reporting higher alcohol consumption (EPI 13.4%, SEPI 5.4%), although the differences among levels of alcohol consumption were not statistically significant (*P* > 0.05). Similarly, cigarette smoking resulted in a higher incidence of EPI and SEPI, while body mass index, diabetes, history of gallstones, or cholecystectomy showed no statistically significant (*P* > 0.05) association with EPI and SEPI. The occurrence of hyperlipidemia was associated with a lower incidence of both EPI and SEPI. Subsequently, several drugs suspected of being associated with pancreatitis and EPI and SEPI were analyzed. It has been observed that in patients receiving angiotensin-converting enzyme (ACE) inhibitors, the incidence of EPI and SEPI was significantly lower than in patients without this drug.

For several years, researchers have been looking for a way to improve the quality of life and health of patients with EPI. Research has been conducted on the use of microbial pancreatic enzymes in pigs to investigate intestinal regeneration and the effectiveness of microbial pancreatic enzyme supplementation on pig performance (Pierzynowski et al., 2012). This supplementation increased the fat and nitrogen absorption coefficient and contributed to an increase in the activity of lipase, amylase, and proteolytic activity, although the values of these coefficients were still lower than in healthy pigs.

## Pancreatic Exocrine Insufficiency

Exocrine pancreatic insufficiency (EPI) causes problems with the digestion of food. EPI prevents the pancreas from producing enough pancreatic enzymes to help the body break down and absorb nutrients. The body cannot efficiently digest and absorb fats, proteins, carbohydrates and vitamins and minerals in food. The damage to the pancreas causing EPI can occur as a result of factors such as: chronic pancreatitis, pancreatic, gastric, or intestinal surgery, cystic fibrosis, Shwachman-Diamond syndrome. Crohn’s disease and celiac disease can also lead to EPI in some people. Also, excessive alcohol consumption is one of the leading causes of chronic pancreatitis. It has been assumed that about 6–12 years of heavy drinking (>80 g of ethanol per day) contributes to the development of chronic pancreatitis caused by alcohol (Dufour and Adamson, 2003). Clinical studies on diabetic patients confirm that this group is particularly susceptible to EPI (Hardt et al., 2000, 2003).

Exocrine pancreatic insufficiency may be asymptomatic initially, but when the damage to the pancreas begins to reduce the capacity to absorb fat, symptoms such as abdominal pain or tenderness, diarrhea, gas, or a feeling of fullness may occur. You can also lose weight and other problems, such as bone pain if you don’t get enough vitamin D, or you can develop a bleeding disorder if you are deficient in vitamin K from not absorbing enough vitamins.

Diagnosis is by taking a blood test to check your body’s vitamin levels and whether your pancreas is synthesizing enough enzymes. It is also good to check the patient for signs of a disease that may lead to EPI (e.g., celiac disease). It may also be necessary to perform a “3-day stool test,” which allows you to check the amount of fat in your bowel movements. Tests such as CT scan, MRI, endoscopic ultrasound are also often performed, which check whether the pancreas is inflamed.

The main treatment for EPI is pancreatic enzyme replacement therapy (PERT). It involves taking tablets with meals that replace the enzymes that the pancreas should produce. These enzymes break down food, making it easier for the body to digest it and absorb the enzymatic digestion of proteins and fats contained in the diet. Sometimes you may also need to take antacids to keep your stomach from breaking down the pancreatic enzymes before they start to work. Administration of a pancreatic polypeptide is a promising new treatment (Ramalho and Dytz, 2020). In addition to taking medications, symptoms can be relieved by following a proper diet. It is recommended to eat six small meals and supplement with vitamins such as A, D, E, and K to replace those not absorbed from the diet (The National Pancreas Foundation, 2021). Alcohol, which makes it difficult for your body to absorb the fat in your food, should be avoided.

Unfortunately, identifying and diagnosing EPI can be difficult as many symptoms overlap with other gastrointestinal disorders.

## Disorders Affecting Pancreatic Functions

Frequently, patients with exocrine pancreatic insufficiency develop other conditions associated with digestive problems. These problems can be both the result of the EPI and its cause (reason). Sometimes they also overlap with the EPI. Therefore, it is important to work closely with your doctor to obtain the correct diagnosis and treatment.

### Inflammatory Diseases

#### Chronic and Acute Pancreatitis

Chronic pancreatitis (CP) is the most common cause of EPI in adults and the most common pancreatic disease. As a result of inflammation, the cells in the pancreas stop working as they should, and the enzymes produced by the pancreas start working before they reach the small intestine. Inflammation in the gland disrupts insulin production, and digestive enzymes may even begin to “digest” the pancreas and surrounding tissues. If left untreated, chronic pancreatitis (CP) can be fatal. From a clinical point of view, several different CP subtypes are distinguished that are well defined in their clinical patterns and morphological imaging features: autoimmune pancreatitis (Finkelberg et al., 2006; de Pretis et al., 2018), paraduodenal pancreatitis (Rebours et al., 2007; de Pretis et al., 2017), pancreatitis associated with gene mutations (Frulloni et al., 2003, 2008).

Basically, we distinguish between two types of inflammation: acute and chronic. Their origin may have many reasons. The risk of inflammation is increased by poor health, genetic predisposition, but most of all by an improper diet and a bad lifestyle (Whitcomb et al., 2016). Many factors can lead to chronic pancreatitis. Some of the most important ones are: alcohol abuse and smoking (Lin et al., 2000), inflammation caused by a malfunctioning immune system, as well as high blood lipids, certain autoimmune disorders, and genetic problems (Merck Manual, 2021). Occasionally, inflammation can occur as a result of certain medications or cancerous changes in the gland. As with many diseases, chronic stress is also one of the main causes of inflammation. Over the years, inflammation can lead to irreversible damage to the pancreas, thereby worsening the digestive processes and damaging insulin-producing cells, leading to the onset of diabetes (Lohr et al., 2017). In addition to digestive system disorders, CP may contribute to developing other complications (resulting from malnutrition) in the cardiovascular system, bones and the immune system (Bang et al., 2014a; Hsu et al., 2016).

Acute pancreatitis occurs unexpectedly without any previous symptoms. The disease is more common in men and usually appears between the ages of 30 and 40. Patients typically complain of lower abdominal and middle back pain, possibly with vomiting, dizziness and increased sweating. If left untreated, CP can cause false cysts to form in the glandular tissue. Once infected, they cause abscesses. In severe cases, there may be blood poisoning, kidney failure, breathing difficulties; eventually, the patient may experience shock. In instances of acute pancreatitis, medications and drips may be necessary. Painkillers, enzyme supplementation, steroid use or, in the case of diabetes mellitus, treatment may be contemplated for chronic inflammation. Diet is necessary for both acute and chronic pancreatitis. In order for the immune system to be active enough to fight inflammation, the intestinal flora must be balanced. The state of the gut flora, otherwise known as the gut microbiome, directly affects the immune system. Animal studies also show that angiotensin-converting enzyme (ACE) inhibitors alleviate chronic pancreatitis and are suggested as a potential treatment for CP (Kuno et al., 2003).

In some cases, the disease results from an inherited disorder of the pancreas or gut. Recurrent acute attacks in people under the age of 30 often turn into a chronic disease. The inherited disease that most often leads to this inflammation is cystic fibrosis. There are also cases where the only possible method of diagnosis is surgery. Hereditary pancreatitis is a progressive disease that may be associated with pain, diarrhea, malnutrition, or diabetes. There are few studies on the long-term survival of patients with CP, and available data indicate that the majority of deaths (60–75%) are due to extra-pancreatic sequelae and possibly alcohol and smoking abuse (e.g., lung or esophagal cancer), liver cirrhosis, and myocardial infarction (Levy et al., 2014). Published studies showed that tuberculosis, gastrointestinal diseases, malignant neoplasms and cardiovascular diseases were the most frequently reported causes of death among CP patients (Yadav et al., 2011; Bang et al., 2014b).

#### Celiac Disease

Celiac disease is a chronic inflammatory bowel disease. People suffering from this condition damage their intestines by consuming gluten (a protein found in wheat, barley, and rye) (Celiac Disease Foundation, 2021). Although the pancreatic exocrine function is normal in this disease, the decreased level of cholecystokinin release (as a result of duodenal villi atrophy) contributes to the impaired gallbladder contraction and reduction of pancreatic function exocrine secretion (Fraquelli et al., 1999; Deprez et al., 2002). Thus, the pancreas may not be secreting enzymes properly due to inflammation and damage to the intestines.

### Neoplastic Diseases

#### Tumors or Cysts

The most serious pancreatic disease is neoplasms, including endocrine neoplasms (pancreatic neuroendocrine tumors) and exocrine neoplasms (pancreatic cancer). Neuroendocrine neoplasms are considered diseases heterogeneous in terms of clinical and pathological features, which have a relatively slow clinical course compared to non-endocrine neoplasms (Rinzivillo et al., 2020). Neuroendocrine tumors (i.e., neuroendocrine neoplasia – NENs) are characterized by a relatively slow growth rate and the ability to secrete various peptide hormones and biogenic amines (Cives and Strosberg, 2018).

Tumors and cysts can cause EPI by blocking the main duct of the pancreas, where enzymes enter the intestines. Pancreatic cancer is resistant to many common treatments, including chemotherapy and radiation therapy. If diagnosed at an early stage, it can be treated surgically. Unfortunately, early diagnosis is rare, and at a later stage, one can only try to improve the patient’s quality of life by treating symptoms and complications alone. Despite many studies, it is still not fully clear what causes pancreatic cancer to appear. Factors that may increase your risk of developing this type of cancer include smoking, diabetes, obesity, chronic pancreatitis, and some hereditary gene mutations. Unfortunately, as many as 95% of pancreatic cancer patients die, even with appropriate treatment.

Pancreatic ductal adenocarcinoma (PDAC) accounts for over 90% of all pancreatic cancers and is the major exocrine tumor subtype (Gloor et al., 2002; Wang et al., 2020). The tumor’s unique microenvironment and its aggressive nature make it poorly responding to conventional chemotherapy, and its poor prognosis translates into an overall 5-year survival rate of 8.5% (Yamamoto et al., 2015). Studies show that the risk of developing pancreatic cancer increases significantly in patients with CP (Hao et al., 2017). Pancreatitis contributes to the development of tumor initiation and progression, influences treatment responses, metastasis and prognosis (Shi and Xue, 2019). Patients diagnosed with CP have an eightfold risk of developing pancreatic cancer 5 years after diagnosis (Kirkegard et al., 2017). Endoscopic ultrasonography (EUS) is the most sensitive, non-surgical method helpful in detecting pancreatic cancer (Luz et al., 2014; Singh and Faulx, 2016).

Pancreatic cysts, in many cases, are detected by computed tomography or MRI obtained during diagnostic tests for a completely different reason. Currently, different types of cysts are diagnosed. Depending on the type of fluid they contain, they are divided into two main varieties. The most common cysts are serous (containing a rare type of fluid) or mucous (containing a thicker, more viscous fluid). Mostly serous cysts are benign and non-cancerous. Most mucous cysts are also benign, although several subtypes may be different in nature. These include: mucinous cystic neoplasm (MCN), which contains ovarian tissue and occurs in women, and endothelial mucosal neoplasm of the main duct (IPMN), a type of mucous cyst that contains many small projections that cover the main pancreatic duct (Wu, 2021).

Currently, the only form of cyst treatment is surgery. However, pancreatic surgery is quite a serious medical undertaking, and therefore resection is performed only in cases where there is a serious risk of tumor formation. In the vast majority of cases, observation with periodic imaging tests is sufficient. Scientists and physicians are continuing their research to determine the best observation interval to monitor these cysts. Clinical studies are also underway to identify more accurate early markers of malignancy.

#### Zollinger-Ellison Syndrome

This rare disorder occurs when growing tumors in the pancreas, duodenum, or part of the small intestine cause the body to release too much of a hormone called gastrin. This, in turn, causes the stomach to release too much acid. This disorder can lead to the appearance of, *inter alia*, stomach ulcers, abdominal pain, and diarrhea, but unlike EPI, the most common symptom is a burning pain between the chest and stomach (National Institute of Diabetes and Digestive and Kidney Diseases, 2021).

### Hereditary Diseases

#### Cystic Fibrosis

Cystic fibrosis is an inherited disease in which there is damage to the pancreatic ducts. It is also the second most common cause of EPI. In patients with cystic fibrosis, pancreatic gland cells break down and become fibrotic. Thickened mucus accumulates in the area of the pancreatic ducts. As a consequence, pancreatic exocrine, and endocrine insufficiency occurs. Adequate nutrition is important in treating cystic fibrosis and EPI. In these diseases, the cells of the lungs and digestive system form thick, sticky mucus, which contributes to the blockage of some organs (Mayo Clinic, 2021). As a result, the pancreas blocks the passage of digestive enzymes into the intestines, where they help break down fats and other nutrients.

#### Diabetes

Currently, diabetes is the third cause of death in the world after high blood pressure and smoking. It is estimated that diabetes affects at least about 8.5% of the population, and this percentage is growing. In order for the human body to maintain the correct level of glucose (blood sugar) within a very narrow range, it needs two hormones: insulin and glucagon, which are secreted by the pancreas (pancreatic endocrine hormones) (Al-Husseina et al., 2021). Frequently, damage from chronic pancreatitis also affects the cells in the pancreas that produce these two hormones. This leads to the development of type 1 or type 2 diabetes (Hardt et al., 2008; Wei et al., 2020). Type 1 diabetes mellitus is an autoimmune disease characterized by the failure of the pancreatic cells to secrete any insulin and leads to a severe increase in blood glucose levels, causing long-term complications (Ma et al., 2018; Turksoy et al., 2018). About 80% of all cases of type 3c diabetes mellitus are associated with chronic pancreatitis (Ewald and Hardt, 2013). However, some recent research suggests that EPI itself can also cause diabetes. The course of the disease is additionally complicated by comorbidities such as poor digestion and accompanying qualitative malnutrition.

Scientists trying to explain the frequent occurrence of exocrine pancreatic insufficiency in diabetes have put forward hypotheses about the dysregulation of exocrine secretion due to diabetic neuropathy (Pirart, 1978) and atrophy of exocrine tissue due to the lack of local trophic action of insulin (Adler and Kern, 1975). The second issue considered was the simultaneous exocrine and endocrine dysfunction and autoimmune inflammation caused by the presence of both specific and exocrine tissue antigens (Kobayashi et al., 1990; Taniguchi et al., 2003) and genetic defects of exocrine endocrine cells (Raeder et al., 2006) (possibly caused by a previous viral or bacterial infection).

Another issue analyzed by the researchers was the assessment of the role of blood pancreatic amylase in the regulation of glucose homeostasis and insulin secretion in a porcine model of streptozotocin- (STZ-) induced diabetes and in a rat pancreatic beta-cell line, BRINBD11 (Pierzynowska et al., 2020). The study shown that amylase supplementation significantly decreased both acute and chronic insulin secretion in the BRIN-BD11 cells. These data confirm the participation of pancreatic amylase in glucose absorption/utilization. Moreover, the direct impact of pancreatic blood amylase on insulin secretion from pancreatic beta-cells and its interactions with insulin and glucagon secretion in a porcine model was observed.

#### Shwachman-Diamond Syndrome

It is an autosomal recessive disease characterized by pancreatic exocrine insufficiency. The clinical features of Shwachman-Diamond syndrome (SDS) include neutropenia, anemia, and thrombocytopenia (Gokce et al., 2013). Other symptoms include recurrent infection tendencies, skeletal changes, and growth failure. This syndrome may contribute to the development of neoplasms in the hematopoietic system (Yamada et al., 2005; Gokce et al., 2013). People with Shwachman-Diamond syndrome may not have the cells in their pancreas that make enzymes.

## Obesity

Obesity is defined as excess body weight resulting from the pathological accumulation of adipose tissue in the body (Marseglia et al., 2015). The main cause of obesity is the disturbance of energy metabolism homeostasis, which results in a positive energy balance leading to weight gain. The World Health Organization (WHO) perceives obesity as a global epidemic, which is not only a significant clinical and social problem in the population of adults, children and adolescents (WHO, 2000; Bryl et al., 2005). Obesity is one of the most important risk factors responsible for excessive deaths worldwide (Flegal et al., 2010; Ahima, 2011; Martinson et al., 2011). Overweight in the developmental age significantly increases the likelihood of this condition in later years and increases the risk of numerous metabolic disorders and negative consequences in adulthood (Morrison et al., 2008; Spiotta and Luma, 2008; Tsigos et al., 2009; Joseph et al., 2011). New research and data from insurance companies show that the health risk of excess fat increases even with relatively small body weight gain, not just severe obesity. The long-term effect of increased body weight at an early age is the development of many chronic diseases, a reduction in the quality of life and reduced efficiency of the body in adulthood. Obesity disease is an unconscious and underdiagnosed disease with serious health consequences.

Obesity is a growing problem in the modern world. Excess body weight affects the development of lipid metabolism disorders, insulin resistance, liver and cardiovascular diseases (at the background of atherosclerosis), hypertension, and sleep apnea. The fat tissue accumulated in the body in the area of the heart, liver, pancreas, and muscles significantly affects the functioning of these organs and poses a significant threat to health (Sweeting, 2007). So far, the influence of excess body weight on the impairment of the exocrine pancreatic function has not been sufficiently studied. Exocrine insufficiency is common in people with type 1 diabetes, about 50% and 30 – 50%, and in some studies, even in 80% of people with type 2 diabetes (Schweiger et al., 2000; Hardt and Ewald, 2011). The carbohydrate disturbances that often accompany obesity correlate with pancreatic exocrine insufficiency. At the American Association for Cancer Research conference in Atlanta (29.03.2019–03.04.2019), it has been shown that overweight and obesity before the age of 50 is more strongly associated with the risk of death from pancreatic cancer than excess body weight later in life. Dr. Eric J. Jacobs et al. analyzed data collected from nearly one million Americans (aged 30–80, cancer-free) enrolled in the American Cancer Society II nationwide survey (American Cancer Society Cancer, 2021). Research carried out in 1982–2014 showed that a higher BMI (Body Mass Index) was associated with an increased risk of death from pancreatic cancer, and that the increase in risk was highest in younger Americans with a higher BMI value. An increase in BMI by five units, i.e., a weight gain of approx. 14.5 kg for a person with a height of ∼ 170 cm, was associated with a 25% increase in the risk of cancer in those who were 30–49 years old at the time of measurement, a 19% increased risk in people aged 50–59, a 14% increase in risk in people aged 60–69, and 13% – in people aged 70–89. According to the researchers, excess body weight may increase the risk of dying from pancreatic cancer much more than previously thought. And unfortunately, it is observed that the last generations are entering the early middle age with a much higher weight than previous generations. The results obtained clearly suggest that in order to stop and reverse the increase in the incidence of pancreatic cancer, one must focus primarily on preventing excessive weight gain in children and young adults (up to 49 years of age). Besides, in addition to preventing pancreatic cancer, it will also help prevent many other diseases (especially civilization diseases).

## Diets

Diet has a huge impact on how a person struggling with pancreatic diseases feels. When the pancreas does not work properly, the human body does not receive enough digestive enzymes to digest and then absorb nutrients from food. Therefore, malnutrition and/or weight loss that is dangerous to human health may occur over time. Especially with pancreatitis, a properly composed diet can relieve abdominal pain associated with this disease. Choosing the right eating plan can put your pancreas at bay and help it recover. It is extremely important and even necessary to switch to an appropriate diet when treating or preventing pancreatic diseases. However, dietary changes do not affect all patients equally. Therefore, it is important to know what foods to eat, what to avoid and how these choices can affect the health of the human body.

A low-fat diet is recommended, which is a modified form of an easily digestible diet (i.e., a diet high in protein, with reduced animal fat, and containing antioxidants). A proper daily energy distribution should consist of: 15% from protein, 20% from fat and 65% from carbohydrates. At the same time, you must provide the body with all the necessary nutrients and vitamins. Recommended fats are mainly vegetable fats: rapeseed and coconut oil, especially oils rich in omega-3 polyunsaturated fatty acids (e.g., olive oil or linseed oil). In moderate amounts, you can use butter 82%. It is necessary to avoid fats containing significant amounts of unsaturated fatty acids containing a double bond of *trans* geometric configuration (except for vaccenic acid). Adding medium-chain triglycerides – fats derived from coconut oil or palm kernel oil – can help improve nutrient absorption in chronic pancreatitis. The available literature confirms the positive effect of supplementation with omega-6 acids (i.e., conjugated linoleic acid isomers) on the fatty acid profile in the pancreas and relative body weight gain (RBWG) of rats (Czauderna et al., 2010).

Due to the limited amount of fat in this diet, deficiencies of macro and micronutrients and vitamins such as calcium, magnesium and zinc, as well as A, D, E, K (soluble only in fats), thiamin and folic acid, may appear (Stigliano et al., 2020). The deficiency of these substances is associated with an increased risk of infections (decreased immunity), cardiovascular diseases, osteoporosis and traumatic fractures (Martínez-Moneo et al., 2016). Therefore, you should increase the amount of fruit and vegetables that are rich in carotenoids. These include most green (i.e., spinach, kale, and lettuce), red and orange (skinless tomato, pumpkin, carrot, peach, apricot, mango, and melon) fruits and vegetables. Antioxidant-rich foods such as dark-leaved vegetables, blueberries, sweet potatoes, grapes, carrots, nuts, and pomegranates are also beneficial. Antioxidants inactivate free radicals appearing in the human body; this helps to reduce the extent of oxidative stress and, at the same time, to reduce inflammation.

You should also avoid fried or high-fat foods, and foods high in sugar and spicy foods. Limiting products such as red meat, offal, French fries and potato chips, mayonnaise, margarine and butter, cakes, sweetened drinks, and full-fat dairy products is recommended. These foods are rich in fats and simple sugars that will increase your triglyceride levels. This increases the amount of fat in your blood and increases your risk of developing acute pancreatitis. Research also suggests that processed meat and red meat increase the risk of pancreatic cancer.

Regularity and the number of meals are very important in this diet. During the day, it is recommended to regularly eat 4–5 meals in small portions. An important feature of the menu, taking into account pancreatic diseases, is the properly balanced distribution of the proportions between protein, fat, and carbohydrates and the way of preparing meals. First of all, the food must be cooked in water or steamed. The vegetables are served mostly cooked. Only juices and purees are allowed raw. You can also use natural medicine. A perfect example of this is a decoction of dandelion root, which supports the activity of the gallbladder and liver, which contributes to the regeneration of the pancreas.

## Life Without a Pancreas

Chronic pancreatitis, emerging tumors (such as adenocarcinoma, neuroendocrine tumors, duodenal cancer or lymphoma), and hyperinsulinemic hypoglycemia (a state of high insulin levels causing a drop in blood sugar levels) may require surgical removal of the pancreas (pancreatectomy). Since there are other organs around the pancreas, the surgeon may also remove: the duodenal intestine (the first part of the small intestine), spleen, part of the stomach, gallbladder, part of the bile ducts. Living without a pancreas is possible, but it requires some important changes. Unfortunately, after surgery to remove this gland, it is necessary to constantly take medications, including drugs that replenish pancreatic enzymes and insulin, and it is also necessary to completely reform the diet.

## Conclusion

The pancreas is an internal organ that plays a key role in the transformation of essential nutrients that provide energy for cells. Problems with its functioning may negatively affect the health of the human body. Even healthy and relatively young people should take care of healthy nutrition and an appropriate lifestyle, which will guarantee optimal pancreatic functioning for many years.

Inappropriate work of the pancreas has a negative impact on the proper functioning of the body, contributing to the development of diseases related to the pancreas, as well as obesity. Likewise, leading a wrong lifestyle and poor diet can cause problems in the proper functioning of this organ. The mere presence of chronic diseases such as obesity may also be a contributing factor to the development of pancreatic disorders.

Obesity disease is an unconscious and underdiagnosed disease with serious health consequences. Adequate diet and adherence to nutritional recommendations, as well as physical activity, as well as psychological support are also elements of the therapy. In the treatment of obesity, the most important thing is to improve the patient’s metabolism, as well as to prevent complications. Obesity is a chronic disease and its treatment should be permanent.

It is possible to manage EPI symptoms by adopting enzyme substitutes and following a nutritional plan that provides adequate nutrition. Eating a diet rich in health-promoting ingredients and replacing pancreatic enzymes with every meal and snack is very important.

A properly composed diet, taking into account the condition of the sick person, can significantly affect the well-being and the course of the disease. Poorly managed diets can lead to interdependent diseases such as diabetes and can even be life-threatening. A pancreatic friendly diet is rich in lean meat protein and low in animal fats and simple sugars.

There is a clear relationship between EPI and age and gender (higher incidence of EPI in men than in women); it can be assumed that, on average, higher consumption of alcoholic beverages, more frequent smoking of tobacco products and less carefully selected health-promoting diet by men compared to women are the reasons for these gender differences.

Most likely, disorders of digestion and food absorption in patients with EPI result in slower growth of the organism, while with age, the influence of exocrine function of the pancreas on organism growth decreases.

## Author Contributions

MK contributed to the conception and design of the study, organized the database, and wrote the first draft of the manuscript. MC provided consultations on the preparation of the work. Both authors contributed to manuscript revision, read, and approved the submitted version.

## Conflict of Interest

The authors declare that the research was conducted in the absence of any commercial or financial relationships that could be construed as a potential conflict of interest.

## Publisher’s Note

All claims expressed in this article are solely those of the authors and do not necessarily represent those of their affiliated organizations, or those of the publisher, the editors and the reviewers. Any product that may be evaluated in this article, or claim that may be made by its manufacturer, is not guaranteed or endorsed by the publisher.
