# Supplementary figures and images for: Pancreas—Its Functions, Disorders, and Physiological Impact on the Mammals’ Organism
Source: Front Physiol. 2022 Mar 30;13:807632. doi: 10.3389/fphys.2022.807632 (PMC9005876; doi:10.3389/fphys.2022.807632)

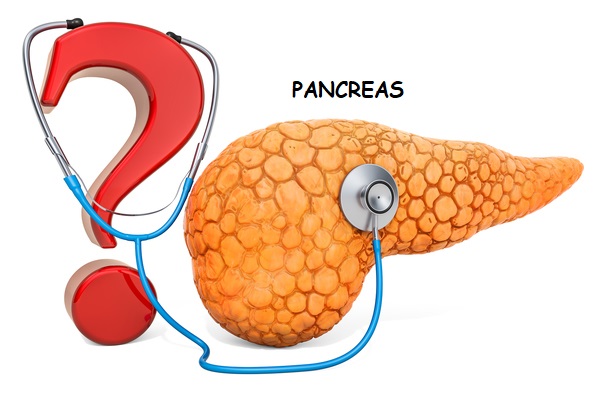

Supplement: Supplementary file 1 [file Image_1.JPEG]
